# The impact of proximity to major central hepatic vasculature on perioperative outcomes and size-based risk stratification in hepatic hemangioma surgery

**DOI:** 10.1371/journal.pone.0332198

**Published:** 2025-09-16

**Authors:** Yulin Xie, Hanrui Yang, Shiqi Lu, Guo Long, Xingyu Mi, Yilin Pan, Hongtao Yuan, Ledu Zhou

**Affiliations:** 1 Department of Liver Surgery, Xiangya Hospital, Central South University, Changsha, Hunan, China; 2 Hepatological Surgery Department, The First People’s Hospital of Guiyang City, Guiyang, Guizhou, China; Tottori University Faculty of Medicine Graduate School of Medicine: Tottori Daigaku Igakubu Daigakuin Igakukei Kenkyuka, JAPAN

## Abstract

**Background and aim:**

Surgical strategies for hepatic hemangiomas remain controversial, particularly concerning the influence of anatomical location and the use of tumor size thresholds. The proximity of tumors to major central hepatic vessels introduces significant surgical complexity and risks, yet its precise effect on perioperative outcomes and the appropriate application of size thresholds under varying anatomical conditions remain understudied. This study aims to evaluate the impact of proximity to major central hepatic vessels on perioperative outcomes and to assess the appropriateness of different size thresholds (8 cm vs. 10 cm) for risk stratification in varied anatomical contexts.

**Methods:**

A retrospective cohort analysis was performed to evaluate hepatic hemangioma patients undergoing surgical resection from October 2016 to July 2024. The collected data included demographics, characteristics of the hemangiomas, laboratory data, surgical approaches, and perioperative variables. Patients were divided into group 1 (non-proximal) and group 2 (proximal) according to major central hepatic vascular proximity.

**Results:**

A total of 309 patients were included in the study, with 176 in group 1 and 133 in group 2.

The perioperative variables including operative duration, Intraoperative Blood Loss, postoperative stay, Intraoperative Transfusion Rate, postoperative complications grade and most postoperative laboratory tests showed significant differences between the two groups (P < 0.05). Group 2(proximal) increased intraoperative blood loss (+139 mL, P = 0.005), operative time (+60 min, P < 0.001), and complication risk (OR=2.44, P = 0.032) versus Group 1(non-proximal). ROC analysis further revealed that the utility of 8 cm and 10 cm as risk stratification thresholds varied significantly depending on tumor proximity.

**Conclusion:**

The proximity of hepatic hemangiomas to major central hepatic vessels significantly elevates surgical difficulty and perioperative risks. Additionally, the sensitivity and specificity of 8 cm and 10 cm as surgical risk stratification thresholds varied distinctly depending on proximity to major central hepatic vessels.

## Introduction

Hepatic hemangioma, the most prevalent benign hepatic tumor with an estimated prevalence of 0.4%−20% [1–3]. Frequently, these lesions are discovered incidentally during imaging studies performed for other medical conditions. For hepatic hemangiomas, conservative management remains the standard of care, as most of these lesions are stable and asymptomatic [4,5]. Consequently, Surgical intervention is reserved for cases with symptoms, risk of complications, or diagnostic uncertainty [6]. Because it is benign, if surgery is advised, a thorough risk assessment is necessary to maximize the surgical approach and timing. Tumor-specific anatomical characteristics, particularly its size and location, are essential to this categorization.

The anatomical location of hepatic hemangiomas considerably determines surgical difficulty and perioperative outcomes. Previous studies have shown a relationship between hemangioma location and perioperative outcomes, such as intraoperative blood loss and postoperative complications [7–9]. Specifically, Yang et al. [10] reported that hemangiomas in specific anatomical regions are situated closer to major intrahepatic vessels. Previous studies [10,11] have confirmed the relationship between vascular proximity and perioperative outcomes in hepatic hemangioma surgery. These findings underline the importance of vascular proximity for surgical risk assessment.

The hepatic hilar region, known for its anatomical complexity and functional importance, is universally recognized as a high-risk surgical area [12]. The first hepatic hilum consists of a complex network of the portal vein, hepatic artery, and bile ducts within Glisson’s capsule. Intraoperative injury in this area can lead to lethal hemorrhage or bile leakage, and tumor compression may cause portal hypertension. Similarly, the second hepatic hilum, located at the junction of the hepatic vein and inferior vena cava, poses surgical challenges because of its thin vascular walls and deep position, increasing the risk of air embolism. Finally, The third hepatic hilum includes fragile short hepatic veins that connect the caudate lobe to the inferior vena cava. These delicate and variable vessels are prone to laceration during dissection, which can lead to uncontrollable retrohepatic hemorrhage. The shared characteristics of anatomical density, structural fragility, and unpredictable variations make hilar injuries more serious than typical intrahepatic vascular damage, and their repair is significantly more complex [13]. Despite these risks, no study has systematically analyzed the impact of major central hepatic vasculature proximity on surgical outcomes.

Furthermore, while tumor size alone is not considered an independent indication for surgery, it does contribute significantly to procedural risk. Historically, previous studies have been discussing the suitability of different size thresholds (such as 8 cm or 10 cm [14,15]) as surgical stratification thresholds.

Given the anatomically plausible hypothesis that proximity to major central hepatic vasculature may critically influence surgical complexity, this study was designed to address two key questions. First, we intended to explore the association between major central hepatic vascular proximity and perioperative outcomes, including intraoperative blood loss, operative time, complication rates, and recovery trajectory. Secondly, we evaluated the predictive performance of 8 cm and 10 cm tumor size thresholds in surgical risk stratification, particularly under different proximity conditions to major central hepatic vessels. By addressing these problems, this research aims to improve surgical planning and risk assessment for patients who need hepatic hemangioma resection, highlighting the critical role of central anatomical relationships in addition to tumor dimensions.

## Materials and methods

### Patients

A retrospective cohort analysis was performed through our institution’s medical records system to evaluate patients with hepatic hemangioma undergoing surgical resection between October 2016 and July 2024. Patients underwent surgical resection based on established clinical indications, including: (1) Significant abdominal symptoms; (2) Documented progressive enlargement; (3) Diagnostic uncertainty where malignancy could not be reliably excluded by imaging; (4) Kasabach-Merritt syndrome; or (5) Significant patient anxiety related to the diagnosis after detailed counseling regarding the typically benign nature, only if this anxiety was documented to cause substantial psychological distress or impaired quality of life upon evaluation. Inclusion criteria required: (1) age 18-80 years; (2) Eastern Cooperative Oncology Group Performance Status (ECOG-PS) score 0-2; (3) Child-Pugh class A/B liver function; and (4) complete preoperative imaging with clinical documentation. Exclusion criteria encompassed: (1) non-hemangioma pathology or concurrent malignancies; (2) history of malignancies (excluding adequately treated non-melanoma skin cancer, cervical carcinoma in situ, and papillary thyroid carcinoma); and (3) prior hepatic interventions including ablation or embolization.

The research protocol obtained approval from the ethics committee of Xiangya Hospital, Central South University (Approval No. 2025040650). The requirement for informed consent was waived because of its retrospective design. Data was accessed for research purposes on 26/04/2025, individuals could not be identified during analyses and evaluations.

### Data collection and definition

The collected data included patient demographics, hemangioma characteristics, hepatitis history, prior upper abdominal surgery, and comorbidities; Laboratory tests from 1 week preoperatively and postoperative days 1–3; Surgical approach; operative duration; intraoperative blood loss; postoperative hospitalization; transfusion requirements and complications.

**Fig 1:**
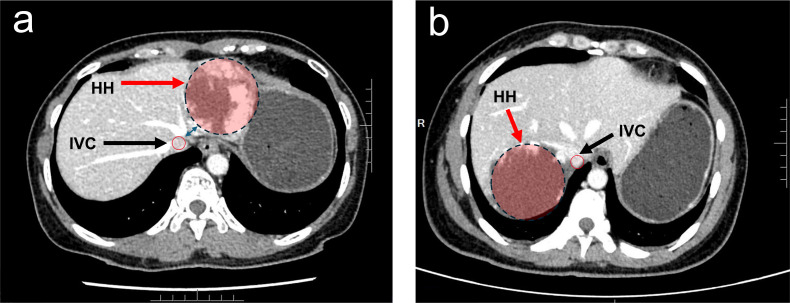
The Radiological Imaging of Proximity to Major Hepatic Vasculature in Hepatic Hemangioma.

All hepatic hemangiomas were preoperatively diagnosed using contrast-enhanced computed tomography (CT) or magnetic resonance imaging (MRI). Radiological assessments evaluated spatial relationships between hemangiomas and major vasculature. Tumor location classification included right lobe, left lobe, and caudate lobe distributions. Proximity to major central hepatic vasculature was defined as ≤1 cm distance from first-order portal vein branches, hepatic venous confluence, or inferior vena cava (IVC) (Fig 1). Patients were divided into Group 1 (non-proximal) and Group 2 (proximal) based on major central hepatic vascular proximity. Within Group 2 (proximal), patients were further subclassified based on the specific central vasculature involved: Subgroup A: proximity to first-order portal vein branches; Subgroup B: proximity to hepatic venous confluence; Subgroup C: proximity to the inferior vena cava (IVC) only; Subgroup D: located in the caudate lobe; Subgroup E: proximity to both first-order portal vein branches and the hepatic venous confluence. Comorbidity assessment incorporated diabetes mellitus, coronary artery disease, and hypertension diagnoses. Surgical approaches were categorized as open or laparoscopic. Intraoperative blood loss was dichotomized as ≥500 ml or <500 ml [16]. Postoperative complications were recorded and graded according to the Clavien-Dindo classification [17], with complications of grade II or higher included in the analysis. Additionally, postoperative liver failure and bile leakage were defined based on the criteria established by the International Study Group on Liver Surgery (ISGLS) [18]. Postoperative mortality was defined as death within 90 days following surgery.

Proximity to major central hepatic vasculature was defined as ≤1 cm distance from first-order portal vein branches, hepatic venous confluence, or inferior vena cava (IVC). The red arrow indicates a hepatic hemangioma within the red zone, while the white arrow identifies the inferior vena cava (IVC).

### Surgical procedures

All surgical procedures in this study were performed by an experienced hepatobiliary surgical team. The choice of surgical approach (open or laparoscopic) was primarily based on tumor size, location, relationship to critical vasculature (e.g., hepatic hilum, hepatic veins), history of prior abdominal surgery, the patient’s systemic condition and preference, with the final decision made by the attending surgeon. Informed consent was obtained from all patients preoperatively. General anesthesia with endotracheal intubation was administered. Patients were positioned supine, with the affected side appropriately elevated based on tumor location to optimize surgical field exposure. The primary resection techniques employed were hepatic hemangioma enucleation and liver resection. Enucleation is the dissection of the tumor along the pseudocapsule or distinct plane between the tumor and the surrounding normal liver parenchyma, whereas liver resection is the removal of the tumor along with a margin of adjacent normal hepatic tissue, which can be anatomical or non-anatomical. The surgeon determined the specific technique intraoperatively based on direct assessment, with procedural safety being the most important consideration.

For laparoscopic surgery, a camera port was established at the umbilicus and CO₂ pneumoperitoneum was induced with pressure maintained at 12–14 mmHg. Surgical trocars were positioned according to tumor location and abdominal domain, typically utilizing five ports arranged in a fan-shaped configuration around the lesion. Laparoscopic exploration was performed, supplemented by laparoscopic ultrasound (LUS) to precisely localize the tumor, delineate its spatial relationship to vascular and biliary structures, and plan the resection pathway. The relevant liver section was fully mobilized to expose the associated hepatic ligaments. A tourniquet for the Pringle maneuver was preplaced around the porta hepatis when indicated. Intermittent portal triad clamping (Pringle maneuver) was routinely applied, with individual clamp durations limited to ≤15 minutes interspersed with 5-minute reperfusion intervals to mitigate hepatic ischemia-reperfusion injury. Under laparoscopic guidance, hepatic parenchymal transection proceeded along the predetermined plane using energy devices such as an ultrasonic scalpel, with meticulous ligation or suture ligation of encountered vessels and bile ducts. A low central venous pressure technique (maintained <5 cm H_2_O) was employed throughout to minimize blood loss.

After the specimen was removed, the surface of the hepatic transection was carefully examined for signs of bile leakage and active bleeding. Hemostasis was routinely achieved using bipolar electrocautery, supplemented by application of absorbable hemostatic agents (e.g., gelatin sponge, hemostatic gauze) as needed. Identified bile duct injuries or leakage points were repaired using Prolene sutures. Drains were positioned next to the transection surface and exteriorized through a trocar site once hemostasis and the lack of bile leakage had been confirmed. The pneumoperitoneum was evacuated and all port sites were closed in layers.

For open surgery, a right subcostal “L” or “J” incision was commonly used. The fundamental procedural steps, such as the Pringle maneuver and parenchymal transection, were similar to those used in the laparoscopic approach. Management of the hepatic transection surface and drainage location followed the same principles.

### Statistical analysis

Continuous variables with normal distribution were expressed as mean ± standard deviation (SD) and analyzed using Student’s t-tests. Non-normally distributed variables were reported as median (interquartile range, IQR) with Mann-Whitney U tests. Categorical variables were presented as frequencies (percentages) and compared using χ² tests or Fisher’s exact tests, as appropriate. Intergroup differences in continuous/ordinal variables were assessed using the Kruskal-Wallis test. For significant overall results (p < 0.05), post-hoc pairwise comparisons were conducted using Dunn’s test. Categorical variables were compared by chi-square tests. If the overall chi-square test was significant (p < 0.05), pairwise z-tests were performed to identify differing subgroups.

Univariate and multivariate logistic regression analyses were conducted to identify risk factors associated with Clavien-Dindo grade ≥ II complications. Additionally, linear regression analyses were performed to examine determinants of intraoperative blood loss and operative duration. Receiver Operating Characteristic (ROC) curve analysis was employed to evaluate the diagnostic value of tumor size thresholds (8 cm and 10 cm) in predicting intraoperative blood loss ≥ 500 ml, operative duration ≥240 min and postoperative complications of grade II or higher across different subgroups. The area under the curve (AUC) was calculated to assess predictive accuracy, and sensitivity and specificity were determined for each threshold.

Statistical significance was set at p < 0.05. Analyses were performed using SPSS 26.0 (IBM, Armonk, NY) and the software R (Version 4.3.2).

## Results

### Patient and hemangioma characteristics

This study included 309 patients undergoing surgical resection for hepatic hemangiomas, categorized into Group 1 (non-proximal to major central hepatic vasculature, n = 176) and Group 2 (proximal to major central hepatic vasculature, n = 133). The cohort had a median age of 50.0 years, with a female predominance (70.9%) and a low prevalence of hepatitis history (89.9% negative). No differences were observed in age, gender, BMI, ASA risk grading, hepatitis, Location, et al between the two groups (P > 0.05). The size of hepatic hemangiomas was significantly larger in Group 2 compared to Group 1 (P < 0.001). Demographic and clinical baseline characteristics are detailed in Table 1. S1 Table introduces the specific indications for hepatic hemangioma surgery and their proportions in each group. There were no statistically significant differences in the distribution of surgical indications between Group 1 and Group 2(X^2^ = 2.97, P = 0.396).

**Table 1:** Comparison of baseline characteristics: non-proximal vs. proximal groups.

Variables	Total (n = 309)	Group 1 (non-proximal) (n = 176)	Group 2 (proximal) (n = 133)	Statistic	*P*
Age(years), M (Q₁, Q₃)	50.00 (40.00, 54.00)	50.00 (41.75, 54.00)	50.00 (39.00, 54.00)	Z = −0.09	0.925
Tumor size(cm), M (Q₁, Q₃)	8.20 (6.60, 10.10)	7.55 (6.20, 9.03)	9.10 (7.40, 11.60)	Z = −5.42	<.001
BMI(Kg/ m^2^), M (Q₁, Q₃)	22.68 (21.15, 24.65)	22.72 (21.23, 24.66)	22.53 (20.96, 24.65)	Z = −0.53	0.598
Gender,n(%)				χ² = 1.44	0.231
male	90 (29.13)	56 (31.82)	34 (25.56)		
female	219 (70.87)	120 (68.18)	99 (74.44)		
Surgical Approaches, n(%)				χ² = 6.18	0.013
Laparoscopic	262 (84.79)	157 (89.20)	105 (78.95)		
Open	47 (15.21)	19 (10.80)	28 (21.05)		
Location, n(%)				χ² = 28.25	<.001
right liver	181 (58.58)	93 (52.84)	88 (66.17)		
left liver	116 (37.54)	83 (47.16)	33 (24.81)		
caudate lobe	12 (3.88)	0 (0.00)	12 (9.02)		
ASA risk grading, n(%)				–	0.667
2	179 (57.93)	103 (58.52)	76 (57.14)		
3	129 (41.75)	73 (41.48)	56 (42.11)		
4	1 (0.32)	0 (0.00)	1 (0.75)		
Priorabdominal surgery, n(%)	15 (4.85)	5 (2.84)	10 (7.52)	χ² = 3.59	0.058
Hepatitis, n(%)	31 (10.03)	15 (8.52)	16 (12.03)	χ² = 1.03	0.310
Comorbidities, n(%)	47 (15.21)	23 (13.07)	24 (18.05)	χ² = 1.45	0.228
Preoperative clinical manifestations, n(%)				χ² = 6.99	0.137
absence of symptoms	215 (69.58)	116 (65.91)	99 (74.44)		
abdominal distension	42 (13.59)	24 (13.64)	18 (13.53)		
abdominal pain	34 (11.00)	23 (13.07)	11 (8.27)		
postprandial fullness	10 (3.24)	9 (5.11)	1 (0.75)		
others	8 (2.59)	4 (2.27)	4 (3.01)		

ASA: American Society of Anesthesiologists, BMI: body mass index.

M: Median, Q₁: 1st Quartile, Q₃: 3st Quartile.

Z: Mann-Whitney test, χ²: Chi-square test, -: Fisher exact.

### Comparative analysis of postoperative parameters in hepatic hemangiomas: Proximal vs. non-proximal to major central hepatic vasculature

Surgical outcomes revealed longer operative durations (243.00 vs. 166.00 minutes, P < 0.001), more Intraoperative blood loss (400.00 vs. 200.00mL, P < 0.001), and higher Intraoperative transfusion rates (20.30% vs. 7.39%, P < 0.001) in Group 2.

Postoperative complications were more frequent in Group 2, with grade ≥2 complications occurring in 27.07% vs. 8.52% (P < 0.001). The incidence of bile leakage was significantly higher in Group 2(14.29% vs. 1.70%, P < 0.001) and open conversion rate were significantly higher (20.95% vs. 4.46%, P < 0.001). Additionally, Group 2 experienced prolonged postoperative hospital stays (6 vs. 5 days, P = 0.008) and worse coagulation profiles, including Postoperative PT(Prothrombin Time) prolongation time (2.13 vs. 1.80 s, P < 0.001) and APTT (2.27vs. 1.30 s, P = 0.004). Furthermore, significant differences were observed between the two groups across most postoperative laboratory parameters. Comprehensive data are presented in Table 2. In the subsequent proximity-based subgroup analysis, intraoperative blood loss (overall P = 0.026) and the platelet count on the first day after the surgery (overall P = 0.009) varied significantly between groups. Tumors in subgroup E(proximity to both first-order portal vein branches and the hepatic venous confluence) were associated with significantly greater intraoperative bleeding (median 600 ml versus 250–450 ml in the other subgroups) and lower the first day platelet counts after the surgery, while all other pairwise comparisons were insignificant. There were no significant differences between subgroups for any other perioperative or laboratory measures, such as operational duration, postoperative length of stay, or open conversion rate (all P > 0.05). Comprehensive data are presented in S2–S4 Tables.

**Table 2:** Perioperative outcomes: non-proximal vs. proximal groups.

Variables	Total (n = 309)	Group 1 (non-proximal) (n = 176)	Group 2 (proximal) (n = 133)	Statistic	*P*
1d albumin(g/L), Mean ± SD	36.16 ± 4.02	36.10 ± 4.24	36.23 ± 3.73	t = −0.27	0.785
3d albumin(g/L), Mean ± SD	34.80 ± 3.25	34.89 ± 3.39	34.68 ± 3.06	t = 0.57	0.566
1d PLT (×10^9^/L), M (Q₁, Q₃)	154.00 (123.00, 185.00)	161.00 (130.25, 192.25)	141.00 (118.00, 173.00)	Z = −3.03	0.002
3d PLT (×10^9^/L), Mean ± SD	149.44 ± 49.66	157.70 ± 51.25	138.51 ± 45.41	t = 3.42	<.001
1d WBC (×10^9^/L), M (Q₁, Q₃)	12.40 (10.10, 14.50)	12.10 (9.90, 14.35)	12.80 (10.50, 14.50)	Z = −1.58	0.115
3d WBC (×10^9^/L), M (Q₁, Q₃)	8.80 (6.70, 11.50)	8.40 (6.60, 10.72)	9.60 (6.99, 12.60)	Z = −2.31	0.021
1d Hb(g/L), M (Q₁, Q₃)	116.00 (105.00, 128.00)	119.00 (109.00,131.00)	111.00 (101.00, 126.00)	Z = −3.59	<.001
3d Hb(g/L), M (Q₁, Q₃)	107.00 (92.00, 119.00)	111.00 (95.67, 122.25)	101.00 (87.00, 115.00)	Z = −4.16	<.001
1d total bilirubin(μmol/L), M (Q₁, Q₃)	18.50 (13.10, 26.00)	18.05 (13.17, 25.02)	18.70 (13.00, 27.40)	Z = −1.04	0.297
3d total bilirubin(μmol/L) M (Q₁, Q₃)	14.80 (10.90, 22.10)	14.70 (10.38, 21.72)	15.00 (11.50, 24.40)	Z = −1.30	0.195
1 d AST(U/L), M (Q₁, Q₃)	321.00 (170.80, 451.00)	270.70 (126.10, 407.08)	370.50 (259.80, 529.20)	Z = −4.43	<.001
3d AST(U/L), M (Q₁, Q₃)	84.40 (46.40, 166.40)	79.40 (35.80, 163.45)	98.90 (59.50, 166.40)	Z = −2.54	0.011
1d ALT(U/L), M (Q₁, Q₃)	284.10 (155.70, 442.60)	244.85 (119.12, 388.80)	344.40 (226.30, 546.90)	Z = −3.97	<.001
3d ALT(U/L), M (Q₁, Q₃)	195.50 (111.80, 327.00)	176.00 (94.00, 285.59)	214.80 (146.20, 360.50)	Z = −2.81	0.005
postoperative hospital stays(day), M (Q₁, Q₃)	5.00 (4.00, 7.00)	5.00 (4.00, 7.00)	6.00 (5.00, 7.00)	Z = −2.67	0.008
Operative Duration(min), M (Q₁, Q₃)	200.00 (145.00, 255.00)	166.00 (125.00, 215.00)	243.00 (200.00, 306.00)	Z = −8.43	<.001
Intraoperative Blood Loss(ml), M (Q₁, Q₃)	300.00 (100.00, 500.00)	200.00 (100.00, 400.00)	400.00 (200.00, 800.00)	Z = −6.14	<.001
Intraoperative Transfusion Rate, n(%)	40(12.94)	13(7.39)	27(20.30)	χ² = 11.21	<.001
Postoperative PT(s), M (Q₁, Q₃)	13.30 (12.21, 14.71)	12.90 (12.07, 14.27)	13.70 (12.80, 15.20)	Z = −3.88	<.001
Postoperative PT prolongation time(s), M (Q₁, Q₃)	1.90 (1.10, 2.70)	1.80 (0.97, 2.41)	2.13 (1.50, 3.30)	Z = −3.42	<.001
Postoperative APTT(s), M (Q₁, Q₃)	28.79 (26.20, 33.20)	28.70 (26.08, 31.77)	29.40 (26.43, 36.50)	Z = −1.86	0.063
Postoperative APPT prolongation time(s),M (Q₁, Q₃)	1.60 (−0.90, 3.53)	1.30 (−1.12, 2.65)	2.27 (−0.70, 5.50)	Z = −2.91	0.004
Open conversion rate, n(%)	29 (11.07)	7 (4.46)	22(20.95)	χ² = 17.39	<.001
Postoperative complications grade ≥2, n(%)				χ² = 18.91	<.001
No	258 (83.50)	161 (91.48)	97 (72.93)		
YES	51 (16.50)	15 (8.52)	36 (27.07)		
Bile leakage, n(%)	22(7.12)	3(1.70)	19(14.29)	χ² = 18.13	<.001

Postoperative PT prolongation time refers to the increase in PT measured after surgery compared to the preoperative baseline value, expressed in seconds.

Postoperative APPT prolongation time refers to the increase in APPT measured after surgery compared to the preoperative baseline value, expressed in seconds.

Postoperative complications were recorded and graded according to the Clavien-Dindo classification.

PLT: Platelet count, WBC: White Blood Cell Count, ALT: Alanine aminotransferase, AST: Aspartate aminotransferase, Hb: hemoglobin, PT: Prothrombin Time, APTT: Activated Partial Thromboplastin Time.

t: t-test, Z: Mann-Whitney test, χ²: Chi-square test, -: Fisher exact, SD: standard deviation, M: Median, Q₁: 1st Quartile, Q₃: 3st Quartile.

1d: the first day after surgery, 3d:The third day after surgery.

### The risk factors associated with Intraoperative Blood Loss In Hepatic Hemangioma Patients

**Fig 2:**
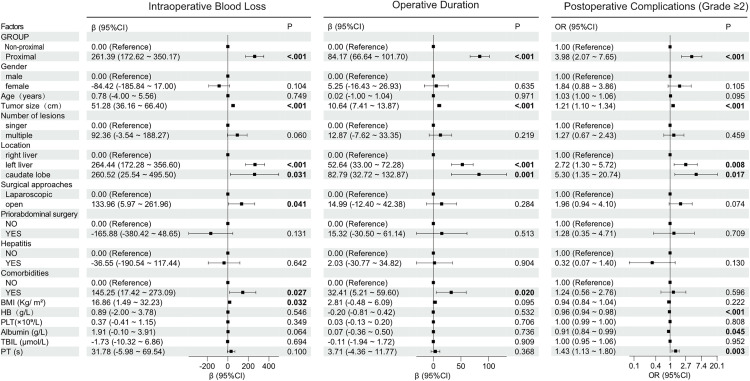
Univariate analysis of intraoperative blood loss, operation duration and postoperative complications (Grade ≥ 2).

**Fig 3:**
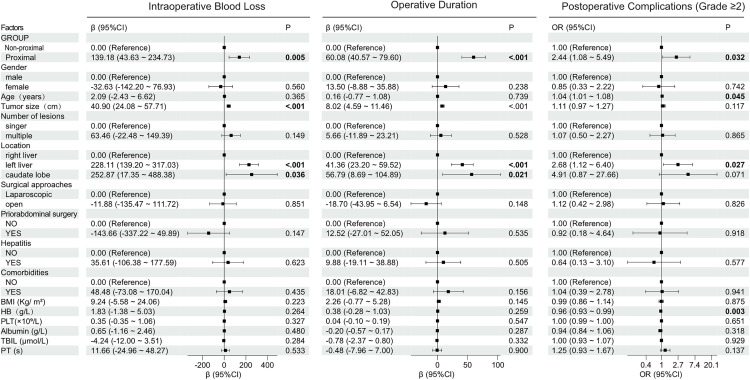
Multivariate analysis of intraoperative blood loss, operation duration and postoperative complications (Grade ≥ 2).

Linear regression models were used to identify factors associated with intraoperative blood loss(IBL)in patients undergoing hepatic hemangioma resection. In the univariate analysis, there were marked differences in BMI, tumor size, location, comorbidities, Group 2 (proximal), open surgery (P < 0.05) (Fig 2). After adjusting for confounders, in the multivariate analysis (Fig 3), Group 2 (proximal) remained independently associated with a 139.18 mL increase in blood loss compared to Group 1 (non-proximal) (β = 139.18, 95% CI: 43.63–234.73, P = 0.005). A 1 cm increase in maximum tumor size continued to predict 40.9 mL higher blood loss (β = 40.9, 95% CI: 24.08–57.71, P < 0.001). Right liver tumors retained significance (β = 228.11, 95% CI: 139.20–317.03, P < 0.001), while caudate lobe tumors showed statistical significance (β = 252.87, 95% CI: 17.35–488.38, P = 0.036).

### Factors associated with prolonged operative duration in hepatic hemangioma resection

Linear regression models were used to identify factors associated with prolonged operative duration in patients undergoing hepatic hemangioma resection. In the univariate analysis, there were marked differences in tumor size, location, Group 2 (proximal) and comorbidities (P < 0.05) (Fig 2). After adjusting for confounders, in the multivariate analysis (Fig 3), Group 2 retained a 60.08 minute increase in operative time (β = 60.08, 95% CI: 40.57–79.60, P < 0.001). Maximum tumor size continued to predict longer duration (β = 8.02 minutes per cm, 95% CI: 4.59–11.46, P < 0.001). Right liver tumors (β = 41.36, 95% CI: 23.20–59.52, P < 0.001) and caudate lobe tumors (β = 56.79, 95% CI: 8.69–104.89, P = 0.021) remained significant predictors.

### Factors associated with postoperative complications (Grade ≥2) in hepatic surgery patients

Logistic regression analysis identified several factors significantly associated with postoperative complications. In the univariate analysis (Fig 2), there were marked differences in Group 2 (proximal), location, tumor size, HB, PT, and albumin(P < 0.05). After adjusting for confounders, in the multivariate analysis (Fig 3), Group 2 remained independently associated with a 2.44 times increased risk (OR = 2.44, 95% CI: 1.08–5.49, P = 0.032). Each additional year of age increased complication risk by 4% (OR = 1.04, 95% CI: 1.01–1.08, P = 0.045). Right liver tumors retained significance (OR = 2.68, 95% CI: 1.12–6.40, P = 0.027), and Higher HB levels remained protective (OR = 0.96, 95% CI: 0.93–0.99, P = 0.003).

### ROC curve analysis of tumor size thresholds

For predicting IBL ≥ 500 ml, the area under the curve (AUC) was 0.64 (p = 0.014) in GROUP1 and 0.72 (p < 0.001) in GROUP2. In Group 1, the > 8 cm threshold achieved a sensitivity of 58% and specificity of 57%, while the > 10 cm threshold showed 32% and 88%, respectively.

**Fig 4:**
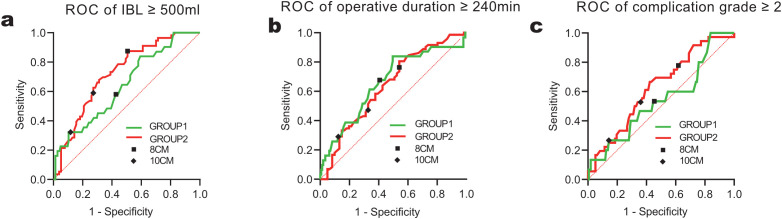
ROC Curve Analysis of Tumor Size Thresholds.

In Group 2, the > 8 cm threshold achieved a sensitivity of 88% and specificity of 49%, while the > 10 cm threshold showed 59% and 73%, respectively. A similar pattern was observed for operation time ≥240 minutes, with AUC values of 0.66 (p = 0.005) in GROUP1 and 0.64 (p = 0.006) in GROUP2. In Group 1, the > 8 cm threshold provided a sensitivity of 68% and specificity of 59%, whereas the > 10 cm threshold yielded 29% and 88%. In Group 2, the > 8 cm threshold provided a sensitivity of 76% and specificity of 46%, whereas the > 10 cm threshold yielded 47% and 67%. For complication grades ≥2, the AUC was 0.54 (p = 0.587) in GROUP1 and 0.63 (p = 0.024) in GROUP2. In Group 1, the > 8 cm threshold had a sensitivity of 53% and specificity of 55%, compared to 27% and 86% for the > 10 cm threshold. In Group 2, the > 8 cm threshold had a sensitivity of 78% and specificity of 38%, compared to 53% and 64% for the > 10 cm threshold. Comprehensive data are presented in Fig 4 and Table 3.

**Table 3:** Predictive Performance of Tumor Size: ROC Curves Analysis in Non-Proximal and Proximal Liver Tumors.

Outcome	Group	Threshold	Sensitivity (95% CI)	Specificity (95% CI)	AUC (95% CI)*	P value*)
Bleeding volume ≥500 ml	Non-proximal	>8 cm	58% (41% to 74%)	57% (49% to 65%)	0.64 (0.54 to 0.75)	0.014
		>10 cm	32% (19% to 50%)	88% (82% to 93%)	–	–
	proximal	>8 cm	88% (76% to 94%)	49% (38% to 60%)	0.72 (0.64 to 0.81)	<0.001
		>10 cm	59% (46% to 71%)	73% (62% to 81%)	–	–
Operation time ≥240 minutes	Non-proximal	>8 cm	68% (50% to 81%)	59% (51% to 67%)	0.66 (0.55 to 0.77)	0.005
		>10 cm	29% (16% to 47%)	88% (81% to 92%)	–	–
	proximal	>8 cm	76% (65% to 85%)	46% (34% to 58%)	0.64 (0.54 to 0.73)	0.006
		>10 cm	47% (36% to 59%)	67% (55% to 78%)	–	–
Complication grade ≥2	Non-proximal	>8 cm	53% (30% to 75%)	55% (48% to 63%)	0.54 (0.39 to 0.70)	0.587
		>10 cm	27% (11% to 52%)	86% (79% to 90%)	–	–
	proximal	>8 cm	78% (62% to 88%)	38% (29% to 48%)	0.63 (0.52 to 0.73)	0.024
		>10 cm	53% (37% to 68%)	64% (54% to 73%)	–	–

## Discussion

The surgical management of hepatic hemangiomas remains clinically challenging. A systematic analysis specifically examining the impact of major central hepatic vascular proximity on perioperative outcomes or its influence on established size thresholds for surgical intervention has been lacking, despite previous research emphasizing the critical role of tumor location in surgical complexity [7,8,10], with some studies [19] advocating for early intervention in cases where hemangiomas are located near major hepatic vasculature and emerging evidence highlighting the importance of tumor-vascular anatomical relationships in guiding surgical decisions [10,11]. Our study addresses this gap by demonstrating that proximity to major central hepatic vasculature significantly increases surgical difficulty and perioperative risks. Furthermore, our findings reveal that the sensitivity and specificity of using 8 cm versus 10 cm as risk stratification thresholds vary across different patient groups based on this proximity. The relatively large sample size increases the generalizability of our findings.

The incidence of conversion from laparoscopic to open surgery is associated with unforeseen intraoperative bleeding or anatomical complexities. Surgery involving hepatic hemangiomas adjacent to major central hepatic vessels requires more intricate anatomical dissection, resulting in increased intraoperative tissue damage and inflammatory responses. Prolonged operative times and higher blood loss further impair postoperative liver function and coagulation system recovery. Despite these surgical risks, no postoperative fatalities were reported in this study. Additionally, the proximity group had larger tumors and a higher prevalence of hemangiomas in the right liver and caudate lobe—factors previously shown to influence perioperative outcomes [7].

To address the impact of baseline differences, we conducted univariate and multivariate regression analyses on key outcomes. Notably, compared to the left liver, locations in the right liver and caudate lobe were significant predictors of both blood loss and operative time (P < 0.05). In the multivariate analysis of postoperative complications ≥ grade II, the P-value for the caudate lobe approached significance (0.07), possibly due to a limited sample size of caudate lobe cases.

In univariate analysis, open surgery was significantly linked with more blood loss than laparoscopic surgery; however, this significance rejected in multivariate analysis, differing from previous studies [20,21]. This disparity may be due to our predisposition to choose open surgery for patients with larger tumors or cases that present higher laparoscopic challenges, which cancels out the effects after controlling for other variables. Tumor size was significantly associated with blood loss and operative time (P < 0.05), consistent with studies indicating that larger tumors necessitate more extensive resection, leading to increased intraoperative blood loss and longer operative times [22,23]. However, while tumor size correlated with a higher risk of complications ≥ grade II in univariate analysis (P < 0.05), this association was not significant in multivariate analysis (P > 0.05). This finding contrasts with some prior studies; Yang [24]reported tumor size as an independent risk factor for complications ≥ grade II, whereas others [23,25] concluded that tumor size is not an independent risk factor. One possible explanation is that our model incorporated variables such as vascular proximity and tumor location, through which tumor size may exert an indirect effect. Larger tumors are more likely to be near major central hepatic vessels or situated in complex regions (e.g., right liver), which increases the risk of complications. When these variables are controlled, tumor size may no longer independently predict outcomes. Additionally, our regression model focused on baseline predictors, whereas other studies included intraoperative variables (e.g., blood loss), which may account for inconsistencies with previous results.

Regarding preoperative laboratory indicators, we found that hemoglobin (HB) was negatively correlated with postoperative complications, aligning with prior study [26]. Low HB indicates preoperative anemia, which correlating with increased perioperative transfusion rates [27]—classified as grade II in the Clavien-Dindo system. Postoperative anemia is associated with poor outcomes including but not limited to infections, increased length of stay, circulatory overload. PT showed a positive correlation with complications in univariate analysis (P < 0.05), possibly reflecting preoperative coagulation abnormalities that elevate postoperative bleeding risk, though this was not sustained in multivariate analysis. A Previous study [28] has not definitively confirmed a link between preoperative PT and postoperative bleeding, warranting further investigation. Through univariate and multivariate regression analyses, this study confirmed that proximity to major central hepatic vessels is an independent risk factor for blood loss, operative time, and postoperative complications ≥ grade II, underscoring its critical role in surgical risk assessment.

Furthermore, the subgroup analysis of the proximal group revealed that significant differences were observed only in intraoperative blood loss and platelet count on the first postoperative day. This was limited to the comparison between the subgroup E(proximity to both first-order portal vein branches and the hepatic venous confluence) and other subgroups, which was likely related to the larger tumor volume and limited operational space in subgroup E. This negative result suggests that for tumors adjacent to vascular structures without simultaneous involvement of both hepatic portals, different central intrahepatic large vessels (portal vein, hepatic vein, or inferior vena cava) may not constitute an independent gradient of perioperative risk. This might also be attributed to the limited sample size.

The precise growth pattern of hepatic hemangiomas remains incompletely understood [2,29,30]. Consequently, determining the optimal timing for intervention is critically important, particularly for patients with asymptomatic progressive growth. Tumor size has consistently served as a significant indicator for evaluating surgical risk. Few prior studies have utilized receiver operating characteristic (ROC) curves to assess threshold appropriateness, especially regarding proximity to different intrahepatic central vessels. For postoperative complications ≥ grade II, no significant correlation was observed in the non-proximity group (P > 0.05), suggesting that tumor size may be a weaker predictor in this cohort, likely attributable to lower surgical complexity. This can be attributed to tumors distant from major central hepatic vessels often being located in peripheral liver segments, which generally present lower surgical risks [9]. For example, hepatic hemangiomas in the left liver are typically treated with laparoscopic left lateral lobectomy, favored for its straightforward exposure and resection [31]. Across all groups, the sensitivity of 8 cm as a surgical threshold consistently exceeded that of 10 cm. This was particularly evident in the proximity group, where sensitivity reached 88% for blood loss >500 ml, indicating its efficacy in identifying high-risk patients. However, the specificity of 8 cm was lower than that of 10 cm; for example, in the non-proximity group with blood loss >500 ml, the specificity of 8 cm was only 57%, potentially resulting in unnecessary surgeries for some patients. In contrast, the 10 cm threshold offered higher specificity (e.g., 88% in the non-proximity group), thereby reducing unnecessary interventions, but its lower sensitivity could result in missing some high-risk cases.

This study emphasize the importance of hepatic hemangioma proximity to major central hepatic vessels in surgical risk assessment, offering valuable guidance for clinical practice. Patients with tumors near major vessels have a higher surgical difficulties and complication risk, needing extensive preoperative imaging evaluations and the consideration of intraoperative hemorrhage control methods. Regarding the application of size thresholds in surgical planning, the applicability of 8 cm versus 10 cm should be balanced based on vascular proximity and clinical objectives: an 8 cm threshold may be preferable for identifying high-risk patients (e.g., proximity group), while 10 cm is better suited to reducing unnecessary surgeries (e.g., non-proximity group). For patients whose tumors are not near major central hepatic vessels, the threshold can be relaxed to 10 cm to minimize unneeded interventions.

However, this study has limitations. Given its retrospective design, there is an inherent risk of selection bias, and full collection and analysis of some specific data, such as biliary duct injury, was not always feasible. Unaccounted confounding factors, such as surgeon experience or specific surgical techniques, could also affect the results. Notably, while multivariate analysis was conducted, it remains challenging to fully account for the inherently different levels of surgical complexity associated with tumors in various locations. Furthermore, this study’s analysis was limited to specific tumor size thresholds of 8 cm and 10 cm for risk stratification, without a broader exploration of other tumor size thresholds. While the overall sample size is relatively large, certain subgroup analyses involved smaller cohorts, potentially limiting the representation of population heterogeneity. Future research could incorporate additional variables (e.g., age, tumor growth rate) to refine surgical decision-making models. Prospective studies comparing long-term outcomes of different surgical thresholds (e.g., 8 cm vs. 10 cm) with conservative management could further optimize individualized treatment strategies.

## Conclusion

This study demonstrates that the proximity of hepatic hemangiomas to major central hepatic vessels significantly elevates surgical difficulty and perioperative risks. Additionally, the sensitivity and specificity of 8 cm and 10 cm as surgical risk stratification thresholds are affected by the anatomical relationship between the tumor and major central hepatic vessels. These findings provide novel evidence for the surgical management of hepatic hemangiomas, highlighting the necessity of personalized assessments based on tumor location.

## Supporting information

S1 Table Indication distribution for hemangioma surgery by group. (DOCX)

S2 Table Comparison of perioperative and laboratory parameters among proximity-based subgroups of hepatic hemangioma. (DOCX)

S3 Table Perioperative outcome rates across proximity-based subgroups. (DOCX)

S4 Table Pairwise comparisons of intraoperative blood loss and platelet count across proximity-based subgroups. (DOCX)

S5 HH clinicalstudy rawdata. (XLSX)
