# Supplementary material for: The impact of proximity to major central hepatic vasculature on perioperative outcomes and size-based risk stratification in hepatic hemangioma surgery
Source: PLoS One. 2025 Sep 16;20(9):e0332198. doi: 10.1371/journal.pone.0332198 (PMC12440192; doi:10.1371/journal.pone.0332198)
Supplement: S1 Table — (DOCX) [file pone.0332198.s001.docx]

**S1 Table. Indication distribution for hemangioma surgery by group**

| Variables | Total (n = 309) | Group 1 (non-proximal) (n = 176) | Group 2 (proximal) (n = 133) | Statistic | *P* |
| --- | --- | --- | --- | --- | --- |
|  |  |  |  |  |  |
| Surgical indications, n(%) |  |  |  | χ²=2.97 | 0.396 |
| Significant clinical symptoms | 94(30.42) | 60(34.09) | 34(25.56) |  |  |
| progressive growth | 199(64.40) | 108(61.36) | 91(68.42) |  |  |
| diagnostic uncertainty | 7(2.27) | 3(1.70) | 4(3.01) |  |  |
| anxiety symptoms | 9(2.91) | 5(2.84) | 4(3.01) |  |  |
