# Supplementary material for: The impact of proximity to major central hepatic vasculature on perioperative outcomes and size-based risk stratification in hepatic hemangioma surgery
Source: PLoS One. 2025 Sep 16;20(9):e0332198. doi: 10.1371/journal.pone.0332198 (PMC12440192; doi:10.1371/journal.pone.0332198)
Supplement: S2 Table — (DOCX) [file pone.0332198.s002.docx]

**S2 Table. Comparison of perioperative and laboratory parameters among proximity-based subgroups of hepatic hemangioma**

| Variables | Subgroup | | | | | *H* | *P* |
| --- | --- | --- | --- | --- | --- | --- | --- |
|  | A (*n*=31) | B (*n*=48) | C (*n*=16) | D (*n*=12) | E (*n*=26) |  |  |
| postoperative hospital stays（day）,M (Q₁, Q₃) | 6.00  (5.00,8.00) | 6.00  (4.25,7.00) | 5.50  (5.00,7.75) | 5.50  (4.00,8.75) | 6.50  (5.00,8.25) | 2.47 | 0.650 |
| Operative Duration（min）,M (Q₁, Q₃) | 243.00  (185.00,280.00) | 231.50  (191.25,280.00) | 255.00  (201.50,363.75) | 273.00  (156.50,332.00) | 265.00  (213.75,352.50) | 4.58 | 0.333 |
| Intraoperative Blood Loss（ml）,M (Q₁, Q₃) | 300.00  (200.00,600.00) | 400.00  (200.00,700.00) | 450.00  (300.00,1150.00) | 250.00  (100.00,600.00) | 600.00  (400.00,825.00) | 11.09 | **0.026*** |
| Postoperative PT（s）,M (Q₁, Q₃) | 13.70  (12.10,14.70) | 13.55  (12.90,14.97) | 13.90  (12.50,15.17) | 13.30  (12.13,15.03) | 14.30  (13.05,16.75) | 4.46 | 0.347 |
| Postoperative PT prolongation time（s）, M (Q₁, Q₃) | 2.30  (1.50,3.00) | 1.95  (1.52,3.25) | 2.50  (1.00,3.92) | 1.75  (0.85,2.15) | 2.40  (1.50,4.83) | 5.79 | 0.215 |
| Postoperative APTT（s）  M (Q₁, Q₃) | 28.20  (25.60,32.40) | 30.60  (27.47,37.52) | 28.10  (26.42,35.88) | 28.25  (26.02,34.35) | 30.95  (26.18,39.95) | 5.27 | 0.260 |
| Postoperative APPT prolongation time（s）, M (Q₁, Q₃) | 1.50  (-1.10,4.70) | 3.55  (-0.18,6.35) | 2.40  (0.13,4.65) | 1.25  (-2.50,2.80) | 1.95  (-0.75,9.55) | 4.49 | 0.344 |
| 1d WBC (×10^9^/L), M (Q₁, Q₃) | 13.10  (10.40,14.50) | 12.00  (9.72,13.73) | 13.25  (10.43,14.80) | 13.10  (11.30,15.15) | 14.05  (10.10,16.48) | 6.96 | 0.138 |
| 1d Hb（g/L）, M (Q₁, Q₃) | 122.00  (104.00,135.00) | 107.00  (98.25,125.75) | 112.00  (87.50,131.75) | 112.00  (106.00,125.00) | 106.50  (98.75,121.25) | 7.19 | 0.126 |
| 1d PLT (×10^9^/L), M (Q₁, Q₃) | 158.00  (130.00,201.00) | 141.50  (116.75,169.00) | 127.00  (122.00,177.50) | 158.00  (142.25,187.50) | 118.00  (104.25,154.25) | 13.65 | **0.009**** |
| 1d albumin（g/L）, Mean ± SD | 38.30  (33.90,40.00) | 36.05  (33.32,38.27) | 35.85  (34.25,39.17) | 36.85  (34.52,38.63) | 35.40  (32.63,38.67) | 5.01 | 0.286 |
| 1d total bilirubin（μmol/L）  M (Q₁, Q₃) | 19.10  (15.00,28.50) | 18.00  (12.27,25.82) | 20.35  (13.05,28.50) | 14.00  (11.60,34.27) | 20.85  (14.97,29.07) | 3.45 | 0.485 |
| 1d ALT（U/L）, M (Q₁, Q₃) | 380.80  (260.90,536.50) | 271.25  (199.32,442.68) | 499.65  (350.88,693.58) | 260.10  (110.25,559.00) | 292.10  (183.32,548.63) | 9.35 | 0.053 |
| 1 d AST（U/L）, M (Q₁, Q₃) | 338.60  (273.20,525.80) | 375.20  (229.10,518.08) | 389.90  (371.10,646.67) | 304.10  (126.33,579.00) | 313.00  (223.85,438.57) | 7.50 | 0.112 |
| 3d WBC (×10^9^/L), M (Q₁, Q₃) | 9.80  (6.70,11.90) | 8.30  (6.53,11.38) | 10.35  (8.55,13.13) | 8.30  (7.03,12.82) | 10.40  (7.45,15.30) | 7.88 | 0.096 |
| 3d Hb（g/L）, M (Q₁, Q₃) | 110.00  (91.00,117.00) | 99.50  (84.50,108.75) | 103.50  (87.00,120.25) | 101.50  (88.25,110.25) | 100.00(  86.25,115.00) | 2.90 | 0.575 |
| 3d PLT (×10^9^/L), M (Q₁, Q₃) | 134.00  (107.00,183.00) | 127.00  (111.00,161.50) | 143.50  (112.50,197.00) | 159.50  (144.75,187.25) | 124.00  (104.00,138.50) | 9.38 | 0.052 |
| 3d albumin（g/L）, Mean ± SD | 34.30  (32.10,36.10) | 33.70  (31.80,35.70) | 35.25  (33.35,38.50) | 35.55  (34.27,36.45) | 34.75  (32.50,37.25) | 7.28 | 0.122 |
| 3d total bilirubin（μmol/L）  M (Q₁, Q₃) | 16.70  (13.60,28.90) | 13.55  (10.93,19.77) | 15.30  (10.00,19.35) | 16.90  (11.93,25.43) | 16.50  (12.15,28.60) | 7.02 | 0.135 |
| 3d ALT（U/L）, M (Q₁, Q₃) | 225.00  (171.10,385.80) | 196.35  (146.93,299.72) | 349.40  (163.95,427.27) | 207.60  (60.42,602.33) | 184.85  (132.43,393.30) | 4.58 | 0.334 |
| 3 d AST（U/L）, M (Q₁, Q₃) | 101.50  (61.00,167.10) | 103.15  (56.13,171.60) | 132.75  (50.83,235.28) | 63.45  (25.35,421.65) | 83.85  (63.25,126.58) | 1.60 | 0.808 |
| Subgroup A: proximity to first-order portal vein branches; Subgroup B: proximity to hepatic venous confluence; Subgroup C: proximity to the inferior vena cava (IVC) only; Subgroup D: located in the caudate lobe; Subgroup E: proximity to both first-order portal vein branches and the hepatic venous confluence. | | | | | | | |
| Postoperative PT prolongation time refers to the increase in PT measured after surgery compared to the preoperative baseline value, expressed in seconds. | | | | | | | |
| Postoperative APPT prolongation time refers to the increase in APPT measured after surgery compared to the preoperative baseline value, expressed in seconds. | | | | | | | |
| PLT: Platelet count, WBC: White Blood Cell Count, ALT: Alanine aminotransferase, AST: Aspartate aminotransferase, Hb: hemoglobin, PT: Prothrombin Time, APTT: Activated Partial Thromboplastin Time. | | | | | | | |
| H: Kruskal-Wallis Test, Q₁: 1st Quartile, Q₃: 3st Quartile. | | | | | | | |
| 1d: the first day after surgery, 3d:The third day after surgery. | | | | | | | |
| * p<0.05 ** p<0.01 | | | | | | | |
