# Supplementary material for: The impact of proximity to major central hepatic vasculature on perioperative outcomes and size-based risk stratification in hepatic hemangioma surgery
Source: PLoS One. 2025 Sep 16;20(9):e0332198. doi: 10.1371/journal.pone.0332198 (PMC12440192; doi:10.1371/journal.pone.0332198)
Supplement: S3 Table — (DOCX) [file pone.0332198.s003.docx]

**S3 Table . Perioperative outcome rates across proximity-based subgroups**

| Variables |  | Subgroup(%) | | | | | Total | Χ^2^ | P |
| --- | --- | --- | --- | --- | --- | --- | --- | --- | --- |
|  |  | A | B | C | D | E |  |  |  |
| Postoperative blood transfusion requirement | NO | 26(83.87) | 38(79.17) | 13(81.25) | 11(91.67) | 18(69.23) | 106(79.70) | 3.19 | 0.527 |
|  | YES | 5(16.13) | 10(20.83) | 3(18.75) | 1(8.33) | 8(30.77) | 27(20.30) |  |  |
| Conversion to open surgery | NO | 20(86.96) | 33(78.57) | 10(71.43) | 9(90.00) | 11(68.75) | 83(79.05) | 3.11 | 0.539 |
|  | YES | 3(13.04) | 9(21.43) | 4(28.57) | 1(10.00) | 5(31.25) | 22(20.95) |  |  |
| Complication grade ≥2 | NO | 23(74.19) | 35(72.92) | 13(81.25) | 8(66.67) | 18(69.23) | 97(72.93) | 1.00 | 0.909 |
|  | YES | 8(25.81) | 13(27.08) | 3(18.75) | 4(33.33) | 8(30.77) | 36(27.07) |  |  |
| Subgroup A: proximity to first-order portal vein branches; Subgroup B: proximity to hepatic venous confluence; Subgroup C: proximity to the inferior vena cava (IVC) only; Subgroup D: located in the caudate lobe; Subgroup E: proximity to both first-order portal vein branches and the hepatic venous confluence. | | | | | | | | | |
| * *p*<0.05 ** *p*<0.01 | | | | | | | | | |
