# Supplementary material for: The impact of proximity to major central hepatic vasculature on perioperative outcomes and size-based risk stratification in hepatic hemangioma surgery
Source: PLoS One. 2025 Sep 16;20(9):e0332198. doi: 10.1371/journal.pone.0332198 (PMC12440192; doi:10.1371/journal.pone.0332198)
Supplement: S4 Table — (DOCX) [file pone.0332198.s004.docx]

**S4 Table . Pairwise comparisons of intraoperative blood loss and platelet count across proximity-based subgroups**

|  | Subgroup | Median | Subgroup | Median | Difference in medians | *p* |
| --- | --- | --- | --- | --- | --- | --- |
| Intraoperative Blood Loss（ml） | A | 300.00 | B | 400.00 | -100.00 | 0.493 |
|  | A | 300.00 | C | 450.00 | -150.00 | 0.168 |
|  | A | 300.00 | D | 250.00 | 50.00 | 0.425 |
|  | A | 300.00 | E | 600.00 | -300.00 | **0.009**** |
|  | B | 400.00 | C | 450.00 | -50.00 | 0.355 |
|  | B | 400.00 | D | 250.00 | 150.00 | 0.184 |
|  | B | 400.00 | D | 600.00 | -200.00 | 0.028* |
|  | C | 450.00 | E | 250.00 | 200.00 | 0.068 |
|  | C | 450.00 | E | 600.00 | -150.00 | 0.397 |
|  | D | 250.00 | E | 600.00 | -350.00 | **0.006**** |
| 1d PLT (×10^9^/L) | A | 158.00 | B | 141.50 | 16.50 | 0.145 |
|  | A | 158.00 | C | 127.00 | 31.00 | 0.214 |
|  | A | 158.00 | D | 158.00 | 0.00 | 0.451 |
|  | A | 158.00 | E | 118.00 | 40.00 | **0.002**** |
|  | B | 141.50 | C | 127.00 | 14.50 | 0.871 |
|  | B | 141.50 | D | 158.00 | -16.50 | 0.067 |
|  | B | 141.50 | E | 118.00 | 23.50 | **0.048*** |
|  | C | 127.00 | D | 158.00 | -31.00 | 0.094 |
|  | C | 127.00 | E | 118.00 | 9.00 | 0.171 |
|  | D | 158.00 | E | 118.00 | 40.00 | **0.002**** |
| Subgroup A: proximity to first-order portal vein branches; Subgroup B: proximity to hepatic venous confluence; Subgroup C: proximity to the inferior vena cava (IVC) only; Subgroup D: located in the caudate lobe; Subgroup E: proximity to both first-order portal vein branches and the hepatic venous confluence. | | | | | | |
| 1d PLT：the platelet count on the first day after the surgery | | | | | | |
| * *p*<0.05 ** *p*<0.01 | | | | | | |
